# The relative age effect in European elite soccer: A practical guide to Poisson regression modelling

**DOI:** 10.1371/journal.pone.0213988

**Published:** 2019-04-03

**Authors:** John R. Doyle, Paul A. Bottomley

**Affiliations:** Business School, Cardiff University, Cardiff, Wales, United Kingdom; Universita degli Studi di Verona, ITALY

## Abstract

Many disciplines of scholarship are interested in the Relative Age Effect (RAE), whereby age-banding confers advantages on older members of the cohort over younger ones. Most research does not test this relationship in a manner consistent with theory (which requires a decline in frequency across the cohort year), instead resorting to non-parametric, non-directional approaches. In this article, the authors address this disconnect, provide an overview of the benefits associated with Poisson regression modelling, and two managerially useful measures for quantifying RAE bias, namely the Indices of Discrimination and Wastage. In a tutorial-like exposition, applications and extensions of this approach are illustrated using data on professional soccer players competing in the top two tiers of the “Big Five” European football leagues in the search to identify paragon clubs, leagues, and countries from which others may learn to mitigate this form of age-discrimination in the talent identification process. As with OLS regression, Poisson regression may include more than one independent variable. In this way we test competing explanations of RAE; control for unwanted sources of covariation; model interaction effects (that different clubs and countries may not all be subject to RAE to the same degree); and test for non-monotonic versions of RAE suggested in the literature.

## Introduction

Relative Age Effects (RAEs) occur when schools and sports group children into age-banded cohorts. Within each cohort, older children study and compete at an artificially conferred advantage over younger children [1–3]. Since the start-points and end-points for age-bandings rarely change, such advantages are conferred consistently throughout childhood. Initial success tends to breed longer-term success for two good reasons. First, there is a *psychological* advantage to being cohort-older. Because it is easy to mistake age-advantage for intrinsic talent, cohort-older children will tend to be nourished on a richer diet of positive regard from teachers, coaches, parents, and peers who see them as more competent than cohort-younger children–as indeed they are [4]. Affirmations from the world tend to become internalised by the child in the form of greater confidence [5] and willingness to engage in competitive activities [6]. Second, there is an *organizational* advantage, in that initial success can also institutionalise success. Specialised training centres, such as football academies, tend to select *young* ‒ when a year can make a big difference ‒ and so their squads grossly over-represent cohort-older children. They also often select *once*, meaning that an academy intake tends to resist future revision. Selecting young and once results in the same large RAE bias propagating from first- to last year in the academy [7–9]. In summary, RAEs are ubiquitous and long-lived into adulthood [1]; they operate by pervasively boosting self-esteem (the *psychological* advantage) and / or by a time-frozen selection bias onto elitist training programs (the *organizational* advantage).

The goal of this paper is to develop, explain and advocate a unified set of analyses that, in Boisot’s [10] terms, can help *codify*, and thus *communicate*, *diffuse*, *accumulate* and ultimately *apply*, knowledge. It is our contention that RAE researchers have not always extracted enough from their data. This limitation occurs both at the local level, in that analyses are often ended prematurely; and at the global level, in that analyses of a present set of results cannot easily be compared with past sets of results that were analysed differently. Perhaps this is to be expected, given that RAE research exists across many disciplines including sports science, education, psychology, health and economics, each with their own traditions of analysis. Although understandable, it is not to be encouraged.

A central place in our framework is occupied by the Poisson regression model (PRM) to analyse the frequency data typical of RAE research. Since many RAE researchers may not be familiar with Poisson regression, we offer a short tutorial introduction structured around substantive research questions raised in the recent RAE literature. Being an “advanced” technique, for an “advanced” audience, PRM is often presented in a way that is inaccessible to the majority—but for digestible overviews, see [11–13]. This is regrettable, as PRM is in fact a relatively straightforward analogue of the popular Ordinary Least Squares (OLS) regression method.

In particular, the PRM shape parameter (the counterpart of the OLS slope) acts within the PRM framework as the common format by which minimally-processed statistical analyses can be translated into more diagnostic but insightful indices and measures, thereby moving RAE research along the Data-Information-Knowledge-Wisdom trajectory [14]. The principal two are: the *Index of Discrimination* (I_D_), which measures the relative odds of someone meeting a criterion given they are born at the start versus end of the cohort year; and two variants of the *Index of Wastage* (I_W1_ and I_W2_), which highlight the degree of talent lost because of RAE. The PRM model is also able to measure the change in RAE, consequent on policy changes to how the cohort year is defined [15].

The empirical analysis is based on an examination of all domestic players in the top two tiers of the “Big Five” European football leagues for the season 2016/17, involving nearly 4000 players. This is more than an illustrative example, but is a proper contribution to the RAE literature. Studies are beginning to emerge using Poisson regression. For instance, Doyle and Bottomley [16] searched, albeit unsuccessfully, for paragon clubs and countries within a European context from which others might learn how to minimise this form of age discrimination. Similarly Brustio et al. [17] documented a diminution of RAE among cohorts of Italian youth as they progressed through the Serie A academy system. Rada et al. [18] raised the interesting possibility that second-tier clubs may provide a second chance for cohort younger players to establish themselves. Sadly, the RAE was just as pronounced among professional players in second-tier clubs as in first-tier clubs in England, France, Germany, Italy, and Spain.

The rest of this paper proceeds as follows. We begin by discussing the basics of Poisson regression modelling and contrast this with the more conventional Ordinary Least Squares approach. Next, we provide a simple illustration with one predictor to the Big Five European football leagues, and show how the Indices of Discrimination and Wastage act as effective measures to summarise RAE bias. We then expand the model with the addition of a second predictor by examining the impact of population birth and death rates, and the challenges posed by multiple cohort year start and end-dates. Finally, we discuss the modelling of interaction terms as the search for paragon clubs, leagues and countries continues [16,18].

## The basics of Poisson regression

Whereas simple bivariate Ordinary Least Squares (OLS) regression finds a best fit to paired (x, y) data of the form:

y=a+bx, (1)

the Poisson regression model (PRM) finds a best fit of the form:

$$y=e^{\left(a+bx\right)}.$$ (2)

Poisson is the default method of analysis when dealing with small expected counts, for instance, the mean number (λ) of unscheduled births a hospital might expect per hour. Students meet the Poisson in “Statistics 101”, where the problem is to build up a profile of the underlying discrete probability distribution (or density function). In this example, the distribution would be the probability of 0 births, 1 birth, 2 births, and so on per hour. Knowing this could help make informed decisions about staffing levels and bed requirements. If instead the window of analysis is one day rather than one hour, then we would expect λ to be 24 times larger as the event is assumed to occur at the same constant rate throughout the interval. Although the distribution is ever more positively skewed for smaller and smaller λ, the Poisson is still the appropriate method.

PRM extends the problem, from λ as a fixed average per unit of time or distance, to situations where λ might vary. The λ governing the expected number of punctures per 100 miles of cycling, for instance, might vary with terrain, speed, tyre pressure, depth of tread, and so on. But at any particular combination of these conditions, there will still be a λ that governs the distribution we might expect; and the Poisson distribution of punctures for that particular λ will look just as it did in Statistics 101. But as the λ for one combination of terrain, speed, and tyres, might be an order of magnitude greater than the λ for another combination, the *scale* and *shape* of each distribution may look very different, though in an entirely lawful and explicable way.

Returning to the algebraic formulation, y = e^(a + bx)^, PRM starts from a given set of x and y and infers the *a* and *b* parameter estimates that best relate them. Just as the intercept, *a*, in OLS shifts the entire regression line up or down, the *a* in PRM does an analogous job by magnifying or shrinking the scale of the entire curve. To see this, notice y = e^(a + bx)^ = e^a^.e^bx^ = *A*e^bx^, with *A* (= e^a^) thereby being the multiplier. Likewise, whereas *b* measures the slope in OLS, the *b* in PRM governs the shape or degree of curvature of the (x, y) relationship. In both OLS and PRM, a positive *b* implies that y increases as x increases; and a negative *b* implies that y decreases as x increases. Statistical tests of the *b* coefficient are tests of the null hypothesis *b* = 0, which translates into a flat horizontal line in both PRM and OLS regression. Given RAE, we anticipate a decrease in frequency across the cohort year, thus *b* should be negative; and the more severe the RAE bias, the more negatively large *b* should be. In the less likely situation of reverse RAE, the *b* should be positive and significant for an underdog effect [19,20]. In what follows, we focus almost entirely on *b*, the Poisson shape parameter.

Just as there is multiple OLS regression, so too can more than one independent variable be included in the PRM estimating equation, and for all the same kinds of reasons. But, one unique feature of PRM not shared by OLS is that for a genuine Poisson process, the mean = variance (= λ), whatever level of λ is observed. The dispersion coefficient is an (aggregated) ratio of variance divided by mean. If dispersion exceeds 1 by a sufficient amount, the likelihood is either that variables are missing from the model which acts to cause more variation than expected, or that the assumptions of Poisson are not being met. The dispersion coefficient is sometimes called the Variance Inflation Factor (VIF) because standard errors of all regression parameter estimates should be scaled by the square root of the VIF to provide more accurate inference, a procedure known as "quasi-Poisson". One corollary is that if dispersion ≈ 1, the assumptions of Poisson are met *and* there are no missing variables that could improve the model. So the ultimate goal of Poisson regression is not to end up with no residual variance (*i*.*e*., R^2^ = 1), as it is in OLS regression. This would result in suspicious levels of under-dispersion, implying that the randomness which underpins the Poisson process had been explained. Instead, the goal is to have residuals in about the “right amounts”, as suggested by a Poisson process. It follows that researchers must keep a careful eye on the dispersion coefficient.

## Poisson application: Big Five European soccer leagues

The data comprise domestic players in the first-team squads of the 204 football clubs competing in the top two tiers of the “Big 5” European leagues (England, France, Germany, Italy, Spain), at the start of season 2016/17, compiled from club websites (see S1 Appendix for complete dataset). Domestic players are defined as those whose nationality matches the league they play in, (e.g., Germans in the Bundesliga). There are 882, 734, 609, 882, and 868 such players in England, France, Germany, Italy, and Spain respectively; in total 3975 of 6644 registered players. This restriction minimises ambiguity due to players learning their trade in a foreign academy, while ensuring that the correct cohort year definition is applied [20]. Players in France, Germany, Italy and Spain are analysed using 1^st^ Jan. to 31^st^ Dec. as the domestic competition (cohort) year, while in England it is defined 1^st^ Sep. to 31^st^ Aug.

How many players were born on each day of the year is the dependent variable (y). The independent variable (x) is time of birth, t_B_, measured as a decimal fraction of the cohort year (0,1), such that players with earlier birthdays have smaller values of t_B_ and the mid-year point is t_B_ = 0.50. Specifically, t_B_ = (*j* ‒ 0.5) / 365, where (*j—*0.5) denotes that players are notionally born at midday on the *j*^*th*^ day of the cohort year, and these values are rescaled by 365 to lie in the one year interval (0,1).

Analysis begins with the overall model, pooled across both countries and tiers, before testing the legitimacy of these assumptions shortly. The best fitting Poisson equation is:

$$Daily\;birth\;frequency=e^{2.7952-0.8786t_{B}},$$ (3)

**Fig 1:**
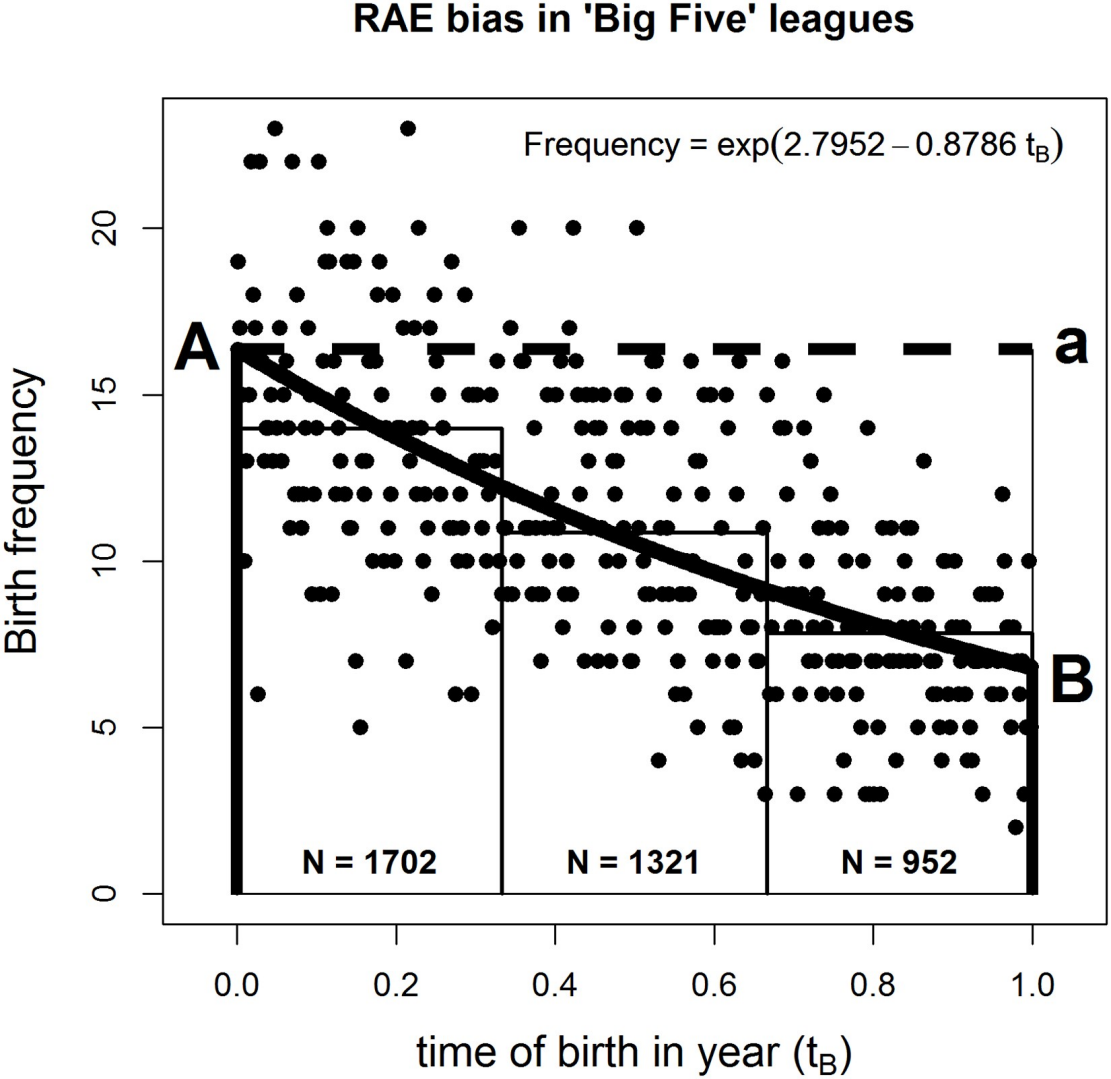
Poisson regression curve with tercile frequencies shown as bars.

And this model explains 40.8% of the variation (McFadden’s pseudo R^2^). The slope coefficient for t_B_ is negative and significantly different from zero (*t* (df = 363) = -15.81, *p* < .001). This shows that the frequency curve trends downwards over the cohort year, consistent with their being more early-born than later-born players, as per RAE. The Poisson *a* coefficient is of little interest. It merely scales the equation to account for the number of observations. Dispersion is 0.985, close to the ideal 1; but all reported significance tests still apply the quasi-Poisson correction. The best fitting model is also presented in Fig 1. From this, we illustrate next the derivation of the Indices of Discrimination and Wastage. These managerially useful, highly interpretable measures, quantify the size of the Relative Age Effect in a standardized way, thereby enabling comparison across future studies.

### Indices of Discrimination and Wastage: A graphical and algebraic understanding

The black curve in Fig 1 shows the best fitting Poisson model. Clearly, players born at the end of the cohort year (point B) have a much smaller chance of reaching the upper echelons of European soccer than players born at the start of the cohort year (point A). The odds of A being selected relative to B is shown by the relative length of the heavy vertical lines projecting to the x-axis from A and B. This is a graphical representation of the Index of Discrimination (I_D_). In algebraic terms, given the Poisson equation *y* = *e*^(*a* + *bx*)^, A occurs when x (= *t*_*B*_) = 0; thus y = p(selected | *t*_*B*_ = 0) = *e*^*a*^. Likewise, B occurs when x (= *t*_*B*_) = 1; thus y = p(selected | *t*_*B*_ = 1) = *e*^(*a* + *b*)^. It follows that:

$$I_{D}=\frac{e^{a}}{e^{\left(a+b\right)}}=\frac{1}{e^{b}}=e^{-b}$$ (4)

Returning to the overall model (Eq 3), and substituting in values for *t*_*B*_ = 0 and *t*_*B*_ = 1 for the selection cut-off dates gives expected player frequencies of 16.37 and 6.80 respectively. Taking the ratio of extremes, the Index of Discrimination (I_D_) = 16.37 / 6.80 = 2.41. Or put more simply, I_D_ is *e*^−*b*^ = *e*^+0.8786^. Thus players born right at the start of the cohort year are nearly two-and-a-half times more likely to reach the upper echelons of professional football than those born right at the end. This I_D_ accords quite closely with Doyle and Bottomley’s [16] 2.67, but their analysis involved 11 nations and was restricted to players in a "top 1000” worldwide listing, as judged by transfer value.

It is important to note that there are several alternative but less precise ways of defining I_D_ which, if ever they entered the RAE literature would immediately undermine the precision of using Poisson regression. In our definition, I_D_ is the ratio of two point estimates which lie at the extreme opposite ends of the cohort year. When seen in this way, forming the ratio of any other pair of points in the cohort year is clearly rather arbitrary (like measuring someone’s height from knee to nose, or from ankle to chin), and would necessarily reduce the size of I_D_. Furthermore, it may be tempting to form a simplified I_D_ ratio, instead, from the numbers born in January versus December; or players in Quarter 1 versus Quarter 4. However, points are no longer being compared, but intervals. And given that such ratios ignore 10 / 12 (months) or 2 / 4 (quarters) of the data respectively, they are particularly vulnerable to sampling fluctuations. So, if researchers wish to make meaningful comparisons across different age groups, countries, sports, times, activities and so on, then it is imperative that they avoid arbitrary redefinitions of I_D_ in favour of a single definition of I_D_.

**Fig 2:**
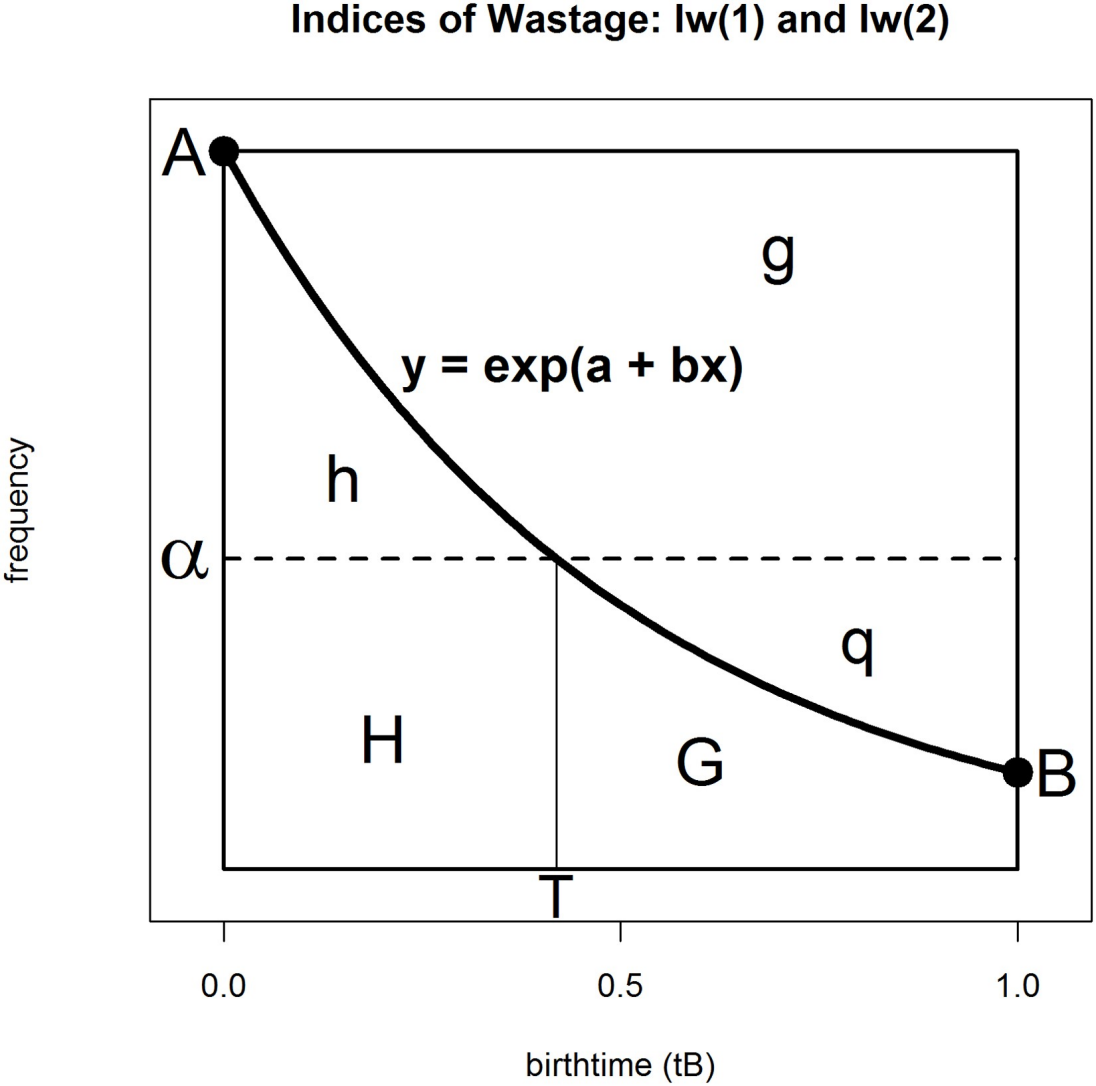
**Indices of Wastage:** I_W1_ and I_W2_.

The fact that *A* players per day born at the start of the cohort year reached a certain criterion suggests that in the absence of RAE, *A* players per day born *anytime* during the year should be able to reach that same criterion. If that were so, in Fig 2 the Poisson curve frequency distribution would run along the horizontal line going through *A*. The totality of players meeting this criterion is given by the area beneath that line. Importantly, this area is partitioned into two sub-areas by the Poisson regression curve. Below the curve are those players the model suggests are currently selected given existing RAE constraints (namely, area h + H + G). In contrast, above the curve are those might-have-beens, the anonymous but equally talented players that remain undiscovered (namely, area g + q). The ratio of undiscovered to discovered areas gives our second diagnostic statistic, the Index of Wastage (I_W1_). It too can be derived algebraically (see S2 Appendix for mathematical details), as follows:

$$I_{W1}=\frac{g+q}{h+H+G}=\frac{1+b-e^{b}}{e^{b}-1}$$ (5)

Returning to the overall model (Eq 3) and substituting in *b* = -0.8786, we find I_W1_ = 0.503. Thus, for every player selected, I_W1_ is the estimated number of players who might have been selected, but have disappeared from the game because they passed through a selection process that rewarded greater maturity in youth. In this instance, for every two talented players who established themselves as professionals, there is one equally talented player that remains undiscovered. In a world without RAE, clubs from the top two tiers could expand their squads by 50%, without sacrificing overall quality. However, this is rather naïve because not only would the wage bill increase dramatically, but players in the now enlarged squads would struggle for game time. Indeed, an even more exaggerated form of squad enlargement would befall football academies, where typically I_W1_ > 1, suggesting that their squads would at least double in size.

Consequently we have developed a second Index of Wastage I_W2_, assuming clubs would not increase squad size, but keep to their same quota that they have in the current RAE-biased world. In Fig 2 maintaining quota amounts to finding a frequency *α* (instead of A), uniform across the cohort year that has the same area under it as the area under the exponential RAE curve. In other words, q + G + H = h + G + H. Immediately we see that q = h. To maintain quota, the number of newly selected cohort-younger players (q) must balance the number of cohort-older players de-selected or “let go”. The mathematical derivation of I_W2_ is a little more involved (see S2 Appendix for details), but can be computed using the following equations:

$$I_{W2}=\frac{q}{G+H}=\frac{q}{\propto -q}$$ (6)

$$\alpha =\frac{A\left(e^{b}-1\right)}{b},\text{where}\;A=e^{\text{α}}$$

$$q=\frac{A}{b}\left(e^{bT}-1\right)-\alpha T,\text{where}\;T=\frac{1}{b}{\text{log}}_{e}\left[\frac{\alpha }{A}\right]$$

Rather more simply, α = N / 365, where N is the number of observations (here, N = 3975 players) in the analysis. For the Big Five leagues data α = 10.89, using either calculation method. Obviously from Fig 2, A > α > B, which it is: 16.37 > 10.89 > 6.80. Likewise T is the point in the cohort year where the horizontal no-RAE dashed line cuts the RAE curve. It marks the boundary between the unfairly advantaged early-borns and the unfairly disadvantaged, later-borns. For the Big Five leagues data, T = 0.4636, which corresponds to the boundary about two weeks (= (0.5–0.4636) * 365 days) before the cohort year mid-point. Finally, I_W2_ = 0.122. Under the new, stricter criterion implied by α, the quota-preserving club would be able to reject h (the least able players born before t_B_ = T) in favour of q (those now retained who would before not have been). The new players (q) as a proportion of the total players selected (G + H + q) accounts for 11% of the players, which can also be calculated as:

$$\frac{I_{W2}}{\left(1+I_{W2}\right)}.$$

While I_W2_ is much smaller than I_W1_ (0.122 versus 0.503) it is nonetheless economically important. Based on I_W2_, out of domestic 3975 players, 437 (= 3975*0.11), around two players per first-team squad (437 / 204) actually have lower skills than another 437 players who are not included in this sample–either they play in lower leagues or gave up their dream of becoming professional footballers altogether–but would have had the potential to reach the top echelons and be included in this sample, had they faced a selection process that took into account their lower maturity in youth. In other words, because clubs rely on a selection process that rewards greater maturity in youth, two players per squad were wrongly selected and could have been replaced by better players to the benefit of owners of soccer clubs, soccer institutions and of course, soccer fans. (We thank our review team for these astute insights).

I_W2_ concerns one-for-one player replacement, with no net loss or gain in player numbers overall. However, I_W1_ concerns pure supplementation, necessarily leading to an increase in overall numbers (but with no players lost). It follows that the kinds of claims and language that can be made based on I_W1_ will not be the same as those based on I_W2_. In fact, I_W1_ and I_W2_ are really just special cases of a more general model in which players can be replaced *and* the overall numbers increased (or even decreased). See S2 Appendix.

To summarise, I_W2_ measures how much more talent we could get out of the football system, assuming we put no more in beyond eliminating RAE. In contrast, I_W1_ measures just how much more untapped talent is out there, with no provisos. The fact that I_W2_ is much smaller than I_W1_ may suggest one reason why clubs persistently ignore RAE. Although there are 2.4 times as many footballers born at the start of the cohort year than at its end (a stark inequality from the player perspective), the gains to the quota-preserving club of removing that inequality are less striking. Just slightly more than two players per squad (who are relatively older) would be replaced (by two relatively younger players).

Together, these indices emphasise the real bias inherent in RAE from different perspectives. This knowledge should also be useful for decision-making purposes where the consequences of RAE bias must be made explicit, and enable comparisons across clubs, tiers, countries, and ultimately time.

Having outlined the benefits of Poisson regression, in the interests of exposition, next we expand the estimating equation by adding a second predictor. Not only will this demonstrate the flexibility of this method compared to conventional chi-squared testing of contingency tables, but it enables us to address pertinent questions raised in the RAE literature, such as the benefits of including data on population births, and the implications of idiosyncratic competition years (e.g., England, Japan).

### Extensions to Poisson regression: Two independent variables

#### Multiple cohort years

For domestic inter-academy games, English clubs define their cohort year as 1^st^ Sept. to 31^st^ Aug. following the academic school year; yet when competing in international club and country competitions they must respect the more usual calendar year definition. It is therefore possible that the calendar year entrains a second RAE bias, albeit more subtle, superimposed on the dominant school year bias; but only for English players. After all, it makes no sense looking for a corresponding September-August effect, in say French or German players, because they will never have competed within that definition of a cohort year when progressing through their youth academy system.

Accordingly, we examined, just for English players, whether T_B_ (Jan.-Dec.) in addition to the domestic t_B_ (Sept.-Aug.) improved the explanatory power of the Poisson regression model. Only the school year (*b*_*1*_ = -0.9360, t(363) = -6.16, *p* < 10^−8^) entrains an RAE bias among English players, and not the calendar year (*b*_*2*_ = -0.0004, t(363) = -1.12, *p* > 0.25). The superiority of the restricted model is confirmed by a model specification *F*-test < 1.

#### Within-year fluctuations in population births

Most RAE studies treat population births as a uniform distribution across the year, an assumption that is only broadly true [21]. While, it is good practice to control for such fluctuations [22], this can be difficult to achieve when datasets span many countries. Nevertheless, deviations from the uniform are much smaller than typical RAE effects, suggesting it is unlikely that they could be solely responsible for any apparent RAE bias.

The UK Office of National Statistics (ONS) has published the number of births in England and Wales for each day of the year during the period 1995–2014. Analysis reveals there is a slight elevation of births during the summer, and somewhat sharper spike in late September / early October, nine months after the Christmas holidays. There are also fewer than expected births on 25^th^ and 26^th^ December suggesting that hospitals can manage this process to some degree.

Overall, ${\bar{t}}_{B}=0.4998\approx 0.5$ when t_B_ is measured using the September to August school year, indicating birth rates are not unfairly delivering an overall boost for or against RAE. For the calendar year, ${\bar{t}}_{B}=0.5032$. While this data does not perfectly align with current football players’ ages, it being too inclusive of more recent years, and Wales (population: 3m) as well as England (53m), it is the best we have found to use as a control variable. Again, when added as the x_2_ variable, population births did not improve model fit beyond the simple one-variable specification of t_B_ alone, *F* < 1.

#### Within-year fluctuations in population deaths

The theory guiding this analysis assumes that whatever seasonal factors increase mortality rates may also bring about ill-health in babies during their critical first months of life. Consequently, such children may be less athletic and have less chance of being selected into elite sporting academies.

ONS publishes detailed statistics about the numbers of deaths on each day of the year, from 1970 through to 2014. Perhaps not surprisingly, there is much more seasonality in deaths than births (coefficient of variation = 0.110 vs. 0.033), peaking when days are shortest and coldest in mid-winter. Having isolated data for 1980–2000 inclusive, we calculated the average number of deaths occurring on each day of the year. This timeframe corresponds to current football players aged 17 to 37 years old. In the event, the second variable is not significant: t(363) = 1.62, *p* > 0.10, and whatever marginal trend there may have been present runs *counter* to our hypothesis.

#### Additional non-linearity

Hjertstrand et al. [23] proposed an “underdog hypothesis”, such that those in the middle of the cohort year gain longer term benefits from having to work harder to keep up with their cohort-older peers, whereas for the youngest, the competition is just too strong. They contend that “those born late–but not too late–will thus end up with the highest skill levels as adults” (p.1). While the Poisson is a non-linear function, a standard way to determine whether there is a discernible bulge or dip relative to the underlying distribution is to add a quadratic term. We found no evidence of acceleration or deceleration for the effect of t_B_ on the Poisson curve, as the coefficient on t_B_^2^ is non-significant and model specification test, *F* < 1. So, while there might be evidence for those born “late but not too late” in the cohort year doing better in terms of educational development, the analogy does not appear to hold with football skills.

Having introduced the notion of a quadratic here, we outline next how to include interaction terms. Although this is slightly more complicated in PRM than in traditional OLS regression, it will likely be important when investigating the generalisability and boundary conditions associated with the RAE phenomena.

### Poisson regression with interaction terms: Clubs, tiers, and leagues

It seems plausible that the intensity of RAE bias may vary between different levels of talent, and in different countries, and for different clubs. There is also a practical side to these investigations, for instance to identify clubs (countries) that may have managed to avoid or attenuate RAE, in order to examine more closely how they have done it. Interestingly, Doyle and Bottomley’s [16] country-by-country analysis for the 11 nations that contributed most professionals to the top 1000 players by transfer value found no evidence of paragon countries. Similarly, their club-by-club analysis of the 32 teams competing in the 2014–15 UEFA Youth Cup, (junior version of the Champion’s League), found no differences in the clubs’ RAE slope coefficients (no club x t_B_ interaction). In a related vein, Rada et al. [18] analysed similar data to that reported here, for the 2014–15 season, to determine whether clubs competing in the second tier of England, France, Germany, Italy, and Spain provided a second chance for later-born players to catch up. But the magnitude of RAE was similar across first and second tiers, both strongly favouring more early-born players. In the interests of generalizability, we conduct a close replication, taking this opportunity to illustrate how interactions are incorporated into the PRM.

Tiers, Leagues and Clubs must be modelled using dummy (factor or category) variables, as all are between-category effects. As such, the data must be disaggregated, not just by days, but by days separately for each Tier. So, the y-variable would be 730 observations, 365 birth frequencies for Tier 1 and 365 birth frequencies for Tier 2, while the x-variables are (i) t_B_ (listed for each Tier), with (ii) a dummy variable for Tier, coded 0 or 1. Similarly, in the case of Leagues, there are 1825 observations (365 days x 5 leagues), and for Clubs there are 74460 observations (365 days x 204 clubs). Clearly, in the latter scenario most of these values will be zero, as the likelihood is that nobody from a particular club will be born on a particular day, but this is not a problem for PRM. The complication arises as the model of interest is *not* just a series of dummies (x_i_), and associated *b*_*i*_ for each of the *i* categories:

$$y=e^{\left(a+a+b1x1+\Sigma bixi\right)},$$ (7)

where x_i_ = 1 for observations belonging to category i, otherwise x_i_ = 0. Instead, we also need a set of interaction terms for each of these dummy variables with x_1_ (= t_B_). Thus:

$$y=e^{\left(a+b1x1+\Sigma bixi+\Sigma \beta i(x1xi)\right)}=e^{\left([a+bixi]+[b1+\Sigma \beta ixi]x1\right)},$$ (8)

The interaction term for t_B_ × Tier indicates how to modify b_1_ to derive Poisson shape parameters for each Tier (League, Club): the second square-bracketed term shows us we add Σβ_i_x_i_. But because of the mutually exclusive nature of the x_i_, we only end up adding a single β_i_ for variable x_i_ which codes for category i: all the other β_j≠i_ get multiplied by corresponding zeros in x_j≠i_.

The results from adding interaction terms were all non-significant: Tiers (*F* < 1), Leagues (*F* < 1), and Clubs (*F* (203, 22817) = 1.104, *p* = 0.15). Full data are provided in S1 Appendix. This is a truly surprising set of results. Once again, there is a main effect for t_B_ indicating that there are more early than later-born players, but the intensity of RAE does not differ systematically between the talented players in Tier 2 and the highly talented players in Tier 1. Nor does RAE differ between the Big 5 leagues–the financially solvent French Ligue 1 and uber-rich English Premier League, where lucrative broadcasting deals *alone* guarantee many clubs return a profit, leading one acerbic media commentator to suggest doing away with ticket sales altogether and playing behind closed doors [24]!

## Discussion and conclusions

The Poisson regression model (PRM), despite being the standard statistical technique for analysing low frequency count data [11–13], remains under-used in Relative Age Effect (RAE) research [16–18]. This tutorial exposition hopes to demonstrate the application and benefits of this approach for this burgeoning research area which is attracting interest from not only those in sports science, but also economics, psychology, education and management. The standard practice is to use non-parametric Chi-squared tests [25] to show that observed quarterly or monthly player frequencies differ from the distribution of a reference population, typically peers that practice the same sport, who are born within the same cohort year. However, should such information be unavailable, for instance, when comparing across large numbers of countries, a uniform distribution may be substituted instead [26].

Yet RAE anticipates a decline in player frequency across the cohort year. Hence, the slope coefficient associated with the Frequency on Birth-time regression forms the basis of two useful and highly interpretable measures, quantifying the magnitude of the Relative Age Effect in a standardized way, namely the Indices of Discrimination (I_D_) and Wastage (I_w_). The I_D_ shows the relative odds of a player born at the *very start* (*t*_*B*_ = 0) of the competition year, compared to a player born on at the *very end* (*t*_*B*_ = 1) being selected. This should not be confused with the ratio of players born in Month 1 versus Month 12 or Quarter 1 versus Quarter 4 or whatever the granularity of the data maybe. Our results suggest that players born right at the start of the cohort year are nearly two-and-a-half times more likely (I_D_ = 2.407) to reach the upper echelons of European professional football than those born right at the end. This accords quite closely with Bottomley and Doyle’s [16] 2.67 based on a subset of players in the “top 1000” from 11 leading nations, and Rada et al.’s [18] 2.13 and 2.22 for comparable analyses of first-tier and second-tier players.

In addition, the Indices of Wastage (I_w1_ and I_w2_) capture the loss of talent because few players are fortunate to be born at the very start of the competition year, and thereby avail themselves of the greater opportunities it provides. I_w1_ measures just how much more untapped talent is out there, with no provisos. Here, I_w1_ = 0.503 which suggests that for every two players identified, there was one equally skilful player that remains undiscovered. In contrast, I_W2_ measures how much more talent we could get out of the football system, assuming we put no more in beyond eliminating RAE. Here I_w2_ = 0.122 which suggests just slightly more than one player per team, or two players per squad (3975 players / 204 teams) who were relatively older would have been replaced by one player who was relatively cohort-younger in an RAE “free world”. Consider only the English Premier League which comprises 20 teams. If one player per team was wrongly selected because they passed through a selection process that rewarded greater maturity in youth, in England there are at least 20 players (about one entire squad) who either play soccer at lower levels or gave up playing soccer altogether but would have had the potential to play in the Premier League and to be better than some current players. Not everybody has the persistence of Leicester City’s Jamie Vardy who was still playing non-league football aged 20. (Again, we thank our review team for these astute observations).

It is only by adopting standardised reporting procedures can we hope to facilitate comparison, build a coherent body of knowledge, and start addressing policy related issues. Unlike analyses based on chi-squared, Poisson regression can *easily* model more than one explanatory variable in the same analysis. For instance, we disentangled the effects of birthtime in the competition year (t_B_) from background variations in the population birth and death rates across the year. We also investigated mixed cohort definitions, as when school year and sports year are different. While our findings remained unchanged, it nevertheless offers a more nuanced understanding of the RAE phenomena, and addresses concerns often raised as limitations in prior studies. With PMR it is also legitimate to perform more disaggregated analyses with smaller samples of data. Thus, we might anticipate witnessing an increase in more micro-analyses, such as club-by-club, academy-by-academy, or even year-group-by-year-group within academy analyses tracking the evolution of the RAE phenomena.

Finally, compared to OLS regression, the modelling of interaction terms is somewhat more complex. But such analyses exploring the conditional nature or contingencies linked to RAE perhaps offers the greatest value added and insights to policy-makers. Presently, there is no evidence to support the existence of paragon clubs, leagues or countries [16,18] from which other organisations may learn. Indeed, there have been few studies exploring the evolution of the RAE phenomena over time [17]. Clearly there is much work to do. Moving beyond another descriptive study documenting the existence of RAE, be it in a different country, competition, cohort, country or time-frame, is paramount. We believe that Poisson regression modelling provides scholars with the necessary tools to do this, and hope you agree!

## Supporting information

S1 Appendix Big 5 leagues dataset. (XLSX)

S2 Appendix Indices of Wastage 1 and 2. (DOCX)

## References

[1] Cobley S, Baker J, Wattie N, McKenna J. Annual age-grouping and athlete development: A meta-analytical review of Relative Age Effects in sport. Sports Medicine. 2009; 39: 235–256 10.2165/00007256-200939030-00005 19290678

[2] Crawford C, Dearden L, Greaves E. The drivers of month-of-birth differences in children’s cognitive and non-cognitive skills. Journal of the Royal Statistical Society, Series A. 2014; 177: 829–860. 10.1111/rssa.12071 PMC4282424 25598586

[3] Matsubayashi T, Ueda M. Relative age in school and suicide among young individuals in Japan: A regression discontinuity approach. PLoS One. 2015; 10: e0135349 10.1371/journal.pone.0135349 26309241 PMC4550458

[4] Hancock DJ, Adler AL, Côté J. A proposed theoretical model to explain relative age effects in sport. European Journal of Sport Science. 2013; 13: 630–637. 10.1080/17461391.2013.775352 24251740

[5] Bai JJ, Mullally K, Soloman D. What a difference a birth month makes: The relative age effect and fund manager performance. Journal of Financial Economics, forthcoming.

[6] Page L, Sarkar D, Silva-Goncalves J. The older the bolder: Does relative age among peers influence children’s preference for competition? Journal of Economic Psychology. 2017; 63: 43–81.

[7] Del Campo DGD, Vicedo JCP, Villora SG, Jordan OR. The relative age effect in youth soccer players from Spain. Journal of Sports Science and Medicine. 2010; 9: 190–198. 24149685 PMC3761747

[8] Deprez D, Vaeyens R, Coutts AJ, Lenoir M, Philippaerts R. Relative age effect and Yo-Yo IR1 in youth soccer. International Journal of Sports Medicine. 2012; 33: 987–993. 10.1055/s-0032-1311654 22791620

[9] Lovell R, Towinson C, Parkin G, Portas M, Vaeyens R, Cobley S. Soccer player characteristics in English lower-league development centres: The relationship between relative age, maturation, anthropometry, and physical fitness. PLoS One. 2015; 10: e0137238 10.1371/journal.pone.0137238 26331852 PMC4558096

[10] Boisot MH. Markets and hierarchies in a cultural perspective. Organization Studies. 1986; 7: 135–158.

[11] Ramsey F, Schaffer D. The statistical sleuth: A course in methods of data analysis. 3^rd^ ed Brooks Cole: Boston, MA; 2013.

[12] Bruner MW, Macdonald DJ, Pickett W, Côté J. Examination of birthplace and birthdate in world junior ice hockey players. Journal of Sports Sciences. 2011; 29: 1337–1344. 10.1080/02640414.2011.597419 21800970

[13] Hayat MJ, Higgins M. Understanding Poisson regression. Journal of Nursing Education. 2015; 53: 207–215. 10.3928/01484834-20140325-04 24654593

[14] Ackoff R. From data to wisdom. Journal of Applied Systems Analysis. 1989; 16: 3–9.

[15] Helsen WF, Starkes JL, Van Winckel J. Effect of a change in selection year on success in male soccer players. American Journal of Human Biology. 2000; 12: 729–735. 10.1002/1520-6300(200011/12)12:6<729::AID-AJHB2>3.0.CO;2-7 11534065

[16] Doyle JR, Bottomley PA. Relative age effect in elite soccer: More early-born players, but no better valued, and no paragon clubs or countries. PLoS One. 2018; 13: e0192209 10.1371/journal.pone.0192209 29420576 PMC5805271

[17] Brustio PR, Lupo C, Ungureanu AN, Frati R, Rainoldi A, Boccia G. The relative age effect is larger in Italian soccer top-level youth categories and smaller in Serie A. PLoS One. 2018; 13: e0196253 10.1371/journal.pone.0196253 29672644 PMC5909613

[18] Rada A, Padulo J, Jelaska I, Ardigò L, Fumarco L. Relative age effect and second-tiers: No second chance for later-born players. PLoS One. 2018; 13: e0201795 10.1371/journal.pone.0201795 30089178 PMC6082547

[19] Gibbs BG, Jarvis JA, Dufur MJ. The rise of the underdog: The relative age effect reversal among Canadian-born NHL hockey players: A reply to Nolan and Howell. International Review for the Sociology of Sport. 2012; 47: 644 649.

[20] Fumarco L, Gibbs BG. Commentary: “How much is that player in the window? The one with the early birthday?” Relative age influence the value of the best soccer players, but not the best business people. Frontiers in Psychology. 2017; 8; Article 58. 10.3389/fpsyg.2017.00058 PMC5263153 28179892

[21] Buckles KS, Hungerman DM. Season of birth and later outcomes: Old questions, new answers. Review of Economics and Statistics. 2013; 95:711–724. 10.1162/REST_a_00314 24058211 PMC3777829

[22] Delorme N, Bioche J, Raspaud M. Relative age effect in elite sports: Methodological bias or real discrimination? European Journal of Sport Science. 2010; 10: 91–96.

[23] Hjertstrand P, Norbäck PJ, Persson L. The educated underdog becomes the ultimate superstar. Research Institute for Industrial Economics, Stockholm. IFN Working Paper, No. 1176. 2017.

[24] Alioa A. Premier League: 11 of 20 clubs could have made profits in 2016–17 without fans at games. BBC Sport website. 14th August 2018.

[25] Delorme N, Champely S. Relate age effect and chi-squared statistics. International Review for the Sociology of Sport. 2015; 50: 740–746.

[26] Schorer J, Cobley S, Brautigam H, Loffing F, Hutter S, Busch D, et al Developmental context, depth of competition and relative age effects in sport: A database analysis and quasi-experiment. Psychological Test and Assessment Modeling. 2015; 57: 126–143.

