# Supplementary material for: The relative age effect in European elite soccer: A practical guide to Poisson regression modelling
Source: PLoS One. 2019 Apr 3;14(4):e0213988. doi: 10.1371/journal.pone.0213988 (PMC6447143; doi:10.1371/journal.pone.0213988)
Supplement: S2 Appendix — (DOCX) [file pone.0213988.s002.docx]

**Appendix S2. Indices of Wastage I_W1_ and I_W2_ and their generalisation.**

**1. Index of wastage, I_W1_ – preserving criterion.**

Talent that gets spotted (*Yes*) corresponds to the area *under* the curve y = e^a+bx^, namely the areas (h + H + g) in the Figure. A criterion is set such that people born at x (= t_B_) = 0 meet the entry level of performance with a frequency of e^a^ (= A). If no RAE bias existed, people born throughout the year would also meet criterion at the same rate as those born at t_B_ = 0. This is signified in the figure by a uniform distribution with height A. It follows that talents missed (*No*) is the area *above* the curve, and below the horizontal line through y = e^a^ (=A), namely the areas (g + q).

I_W1_ is the ratio of missed to spotted, No / Yes, given the assumption that the criterion level of performance that accepts those born at t_B_ = 0 applies across the cohort. In theory, whether any particular individual meets the criterion could be established on that person’s N^th^ birthday, thus removing relative age as a confound of talent.

Yes = h + H + G = $\int_{0}^{1} e^{a+bx}dx$ = $e^{a}\int_{0}^{1} e^{bx}dx$ = $e^{a}\left[\frac{e^{bx}}{b}\right]_{0}^{1}$ = $e^{a}\left[ \frac{e^{\mathrm{bx}}}{b} \right]_{0}^{1}$

But, Yes + No = Area of rectangle (all those sufficiently talented) = e^a^ . (1 – 0)

So, No = $e^{a}$– Yes = $e^{a}\left( 1- \frac{1}{b}\left( e^{b}-1 \right) \right)$

Therefore **I_W1_ =** (No / Yes) =  $\frac{(1+b- e^{b})}{(e^{b}-1)}$**, or $\frac{b}{(e^{b}-1)}-1$.**

Note, as b → 0, both numerator and denominator of the I_W1_ ratio → 0, but we can use l'Hôpital's rule to show that $\frac{b}{(e^{b}-1)}$ → $e^{-b}$ → 1. Thus, as b → 0 (*i.e.,* Poisson curve is horizontal), I_w1_ → 0, as desired.

To give an example, if I_W1_ = 2 among academy players in a particular club, it means that for every $\frac{b}{(e^{b}-1)}-1$player in the academy, that club could have recruited two additional players capable of reaching the same standard as those born at t_B_ = 0. Effectively, they could have tripled their player base by eradicating RAE.

But to accommodate these extra players, there would be obvious financial and logistical implications. Only so-called “selling clubs” might be tempted by this model, hoping to gain revenue from their excess talent. However, to prevent flooding the labour market, clubs adopting this strategy would have to be in the minority. Yet even well-known selling clubs such as Ajax seem, like everyone else, to have squads constrained by the requirements of running a single team of eleven players plus substitutes. In the context of club academies, then, I_W1_ may be a slightly naïve yardstick as to what can be achieved by eliminating RAE, and in the next subsection we next develop a second perspective on talent wastage, I_W2_. Instead of eliminating RAE by holding talent criterion constant and expanding quotas, we hold quotas constant but alter criterion.

Nonetheless, I_W1_ has its uses. At the country level I_W1_ *is* a natural and appropriate measure to discuss policy implications of the nation’s talent. This paper examines the top two tiers of the professional game in five countries. As a pertinent example therefore, suppose I_W1_ = 0.5, it follows that with creative policies to eliminate RAE, that country could have filled the top *three* tiers of its leagues with the same overall quality of (domestic) players that currently occupy tiers 1 and 2. This simple insight based on I_W1_ is intelligible, uncontentious, and leads us on to think about consequences.

**2. Index of wastage, I_W2_ – preserving quotas.**

If a club manages to eliminate RAE, but wishes to maintain the same quota of players per year as before, then the frequency of selection is α, uniformly across the year, as shown.

I_W2_ = $\frac{q}{H+G}$

But because the quotas are the same with and without RAE, the area beneath the dotted line rectangle (without RAE) equals the area under the exponential curve (with RAE). Also, those now selected who previously were not (q) must balance those previously selected, but who no longer are (h), so that h = q.

Hence, α . 1 = H + G + h = H + G + q, so that the useful form for I_w_(2) is:

I_W2_ = $\frac{q}{\alpha-q}$ (λ)

But α = *Yes* as already calculated in the previous section for I_W1_. So we need only to calculate q, which we do by first calculating (h + H), then calculating H, and subtracting to leave h (= q), as desired.

H + h = $\int_{0}^{T} e^{a+bx}dx$ = $e^{a}\int_{0}^{T} e^{bx}dx$ = $e^{a}\left[\frac{e^{bx}}{b}\right]_{0}^{T}$ = $e^{a}\left[ \frac{e^{\mathrm{bx}}}{b} \right]_{0}^{1}$ = $\frac{A(e^{bT}-1)}{b}$

α = $e^{a+bT}$ = *Ae^bT^*

T = $\frac{1}{b}\log_{e}\left(\frac{\alpha}{A}\right)$

H = αT

Hence q = h = (h + H) – H

q = $\frac{A}{b}(e^{bT}-1)-\alpha T$.

An alternative derivation and formula is:

q = (G + q) ‒ G = α(1 – T) ‒ $\int_{T}^{1} Ae^{bx}dx$

= α(1 – T) ‒ $\frac{A}{b}(e^{b}-e^{bT})$

Now knowing both α and q, we can determine I_W2_ from the above equation marked (λ).

Using values for b in the range [0, -3], we estimate the following regression which shows how the ratio I_W1_ / I_W2_, varies dependent on b:

$\frac{I_{W1}}{I_{W2}}$ = 4.002057029 ‒ 0.157743621 b ‒ 0.023551504 b^2^; R^2^ = 0.99963.

**3. Hybrid model: supplementation (as in I_W1_) and replacement (as in I_W2_).**

**
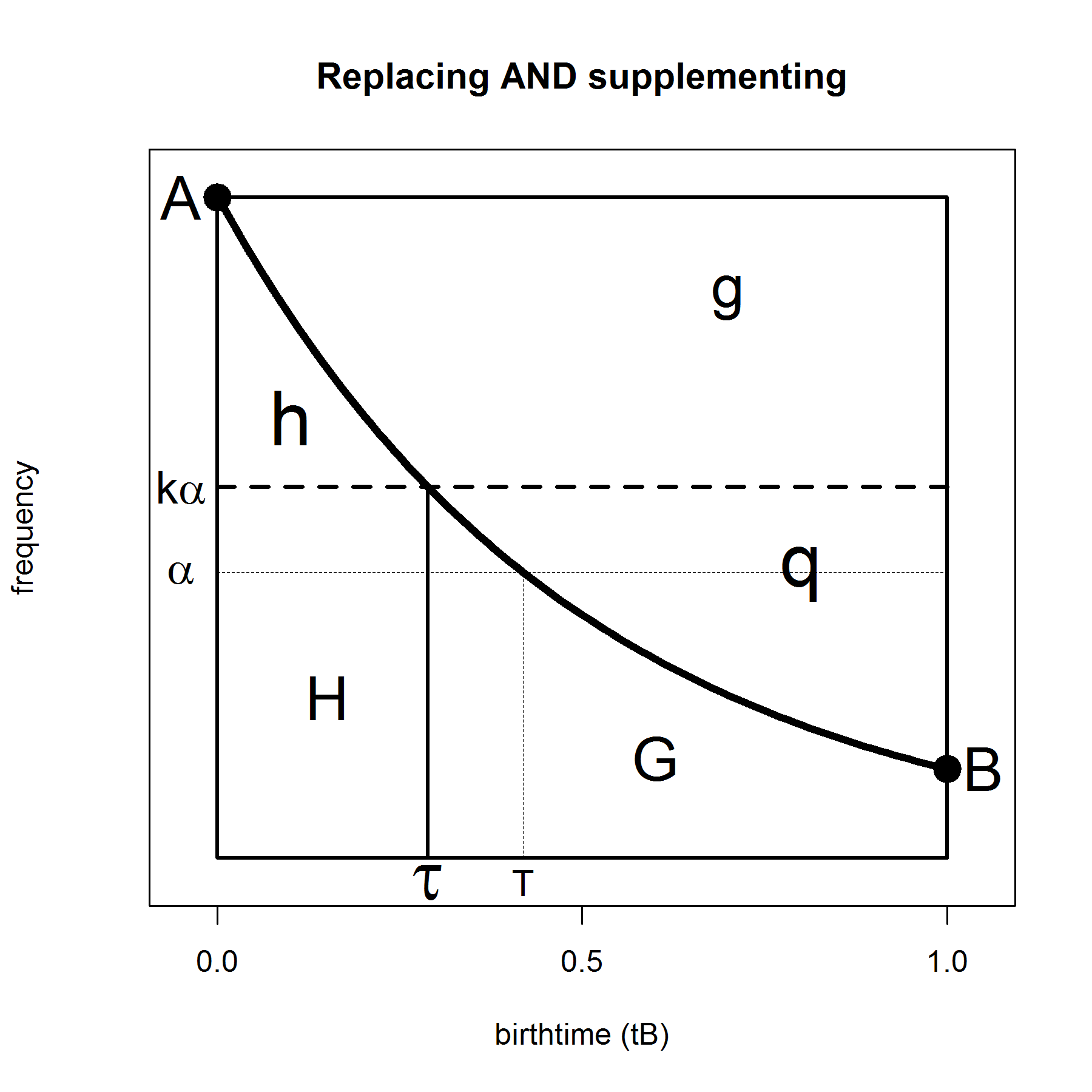
**

Figure. Note, subareas h, H, G, q, and g are defined by the horizontal (dashed) line at kα and, the vertical line at τ, and the Poisson curve. The faint lines at α and T reference the previous section.

Suppose clubs expand (contract) the size of their academies by a factor k, this is equivalent to moving the horizontal dashed line at α towards A, as shown in the above figure to kα (where a = 0.5, b = -1, and k = 1.3 for a 30% expansion of academies). We do not want this new line to exceed A or to fall below B, therefore: $\frac{B}{\alpha} \leq k \leq\frac{A}{\alpha}$. Following a similar process of adding and subtracting areas, already used to determine I_W2_, we can obtain formulae for the subareas h, H, G, q, g, together with their derivatives with respect to k.

| $A= e^{a}$ | $A^{'}= \frac{dA}{dk} = 0$ |
| --- | --- |
| $\alpha=\frac{A(e^{b}-1)}{b}$ | $\alpha^{'}= \frac{d\alpha}{dk}=0$ |
| $\tau=\frac{1}{b}{log}_{e}\left( \frac{k\alpha}{A} \right)$ | $\tau^{'}= \frac{d\tau}{dk}= \frac{1}{bk}$ |
| $H=k\alpha\tau$ | $H^{'}= \frac{dH}{dk}= \alpha\left( \tau+ \frac{1}{b} \right)$ |
| $h=\frac{A}{b}\left( e^{b\tau}-1 \right)-H$ | $h^{'}= \frac{dh}{dk}= -\alpha\tau$ |
| $G=\frac{A}{b}\left( e^{b}-e^{b\tau} \right)$ | $G^{'}= \frac{dG}{dk}= -\frac{\alpha}{b}$ |
| $q=\left( 1-\tau\right)k\alpha-G$ | $q^{'}= \frac{dq}{dk}= \alpha\left( 1- \tau\right)$ |
| $g=A-\left( h+H+G+q \right)$ | $g^{'}= \frac{dg}{dk}= \alpha\left( \tau-1 \right)$ |

Using these components, many different new indices may be created. Here, we pick out just the replacement ratio: $\frac{h}{q}$. The following table uses illustrative values of k, with a = 0.5, and b = -2, as in the Figure. Note, $\frac{A}{\alpha}$ = 2.3130, which is the upper bound for k.

|  |  |  |  |  |
| --- | --- | --- | --- | --- |
| k | 1 | 1.3 | 1.5 | 2.3130 |
|  |  |  |  |  |
|  |  |  |  |  |
|  |  |  |  |  |
| A | 1.6487 | 1.6487 | 1.6487 | 1.6487 |
| α | 0.7128 | 0.7128 | 0.7128 | 0.7128 |
| τ | 0.4193 | 0.2881 | 0.2165 | 0.0000 |
| H | 0.2989 | 0.2670 | 0.2315 | 0.0000 |
| h | 0.1691 | 0.0941 | 0.0582 | 0.0000 |
| G | 0.2448 | 0.3518 | 0.4230 | 0.7128 |
| q | 0.1691 | 0.3079 | 0.4146 | 0.9359 |
| g | 0.7668 | 0.6280 | 0.5213 | 0.0000 |
|  |  |  |  |  |
| **h/q** | **1.0000** | **0.3055** | **0.1404** | **0.0000** |

Looking at the h/q ratio (exits to entries), when k = 1, we have one-for-one substitution (implied by I_W2_, where h = q by definition). At the other limiting value of k (= 2.3130 = A/α) there are no exits, only entries: a strategy of pure supplementation (implied by I_W1_). For values of k in between, there is net supplementation, but also substitution.

Finally, the generalised index of wastage is $I_{W*}= \frac{q}{G+H}$. When kα = A, I_W*_ = I_W1_; when k=1, I_W*_ = I_W2_; and when kα = B, I_W*_ = 0.
